# Spring migration patterns, habitat use, and stopover site protection status for two declining waterfowl species wintering in China as revealed by satellite tracking

**DOI:** 10.1002/ece3.4174

**Published:** 2018-05-24

**Authors:** Yali Si, Yanjie Xu, Fei Xu, Xueyan Li, Wenyuan Zhang, Ben Wielstra, Jie Wei, Guanhua Liu, Hao Luo, John Takekawa, Sivananintha Balachandran, Tao Zhang, Willem F. de Boer, Herbert H. T. Prins, Peng Gong

**Affiliations:** ^1^ Ministry of Education Key Laboratory for Earth System Modeling Department of Earth System Science Tsinghua University Beijing China; ^2^ Resource Ecology Group Wageningen University Wageningen The Netherlands; ^3^ Department of Animal and Plant Sciences University of Sheffield Sheffield UK; ^4^ Department of Ecology and Evolutionary Biology University of California Los Angeles California; ^5^ Naturalis Biodiversity Center Leiden The Netherlands; ^6^ Jiangxi Poyang Lake National Nature Reserve Authority Jiangxi Poyang Lake Wetland Ecosystem National Research Station Nanchang China; ^7^ Suisun Resource Conservation District Suisun City California; ^8^ Bombay Natural History Society Mumbai India; ^9^ Joint Center for Global Change Studies Beijing China

**Keywords:** *Anser albifrons*, *Anser serrirostris*, habitat selection, protected area, stopover site

## Abstract

East Asian migratory waterfowl have greatly declined since the 1950s, especially the populations that winter in China. Conservation is severely hampered by the lack of primary information about migration patterns and stopover sites. This study utilizes satellite tracking techniques and advanced spatial analyses to investigate spring migration of the greater white‐fronted goose (*Anser albifrons*) and tundra bean goose (*Anser serrirostris*) wintering along the Yangtze River Floodplain. Based on 24 tracks obtained from 21 individuals during the spring of 2015 and 2016, we found that the Northeast China Plain is far‐out the most intensively used stopover site during migration, with geese staying for over 1 month. This region has also been intensely developed for agriculture, suggesting a causal link to the decline in East Asian waterfowl wintering in China. The protection of waterbodies used as roosting area, especially those surrounded by intensive foraging land, is critical for waterfowl survival. Over 90% of the core area used during spring migration is not protected. We suggest that future ground surveys should target these areas to confirm their relevance for migratory waterfowl at the population level, and core roosting area at critical spring‐staging sites should be integrated in the network of protected areas along the flyway. Moreover, the potential bird–human conflict in core stopover area needs to be further studied. Our study illustrates how satellite tracking combined with spatial analyses can provide crucial insights necessary to improve the conservation of declining Migratory species.

## 1 INTRODUCTION

Migratory birds are essential indicator species for monitoring ecosystem health (Bauer & Hoye, 2014; Steele, Bayn, & Grant, 1984). They can alter the composition of resident communities and change ecosystem functioning by transporting nutrients and organisms at local, regional, and global scales (Altizer, Bartel, & Han, 2011; Bauer & Hoye, 2014; Si et al., 2009). Migratory birds can travel vast distances in a relatively short time and exploit the seasonal food surplus in the high Arctic during summer, while avoiding scarcity during the harsh Arctic winter by returning to temperate or tropical areas (Newton, 2008; Somveille, Rodrigues, & Manica, 2015). Bird migration is therefore predictable, as migrants tend to synchronize their spatial distribution with the seasonal availability of food resources (Drent, Fox, & Stahl, 2006; Si, Xin, de Boer, et al., 2015). Synchrony between distribution and resources also makes migratory birds particularly vulnerable to alterations in food availability along the flyway by global climate and land cover change (Drent et al., 2007; Knudsen et al., 2011; Si, Xin, Prins, de Boer, & Gong, 2015; Van Eerden, Drent, Stahl, & Bakker, 2005). Hence, understanding the ecology of bird migration is crucial for the conservation of migratory species.

Among the global network of migratory waterfowl flyways, the Asian flyways are the least studied in terms of migration ecology, while they are characterized by the most pronounced human–bird conflicts (Si, Xin, Prins, et al., 2015). East Asian migratory waterfowl have greatly declined since the 1950s, and several species are at critically low numbers (Cao, Barter, & Lei, 2008; de Boer et al., 2011; Syroechkovskiy, 2006). Although hunting remains a problem, habitat loss and degradation of stopover and wintering sites, especially those in China, are considered the main threat (de Boer et al., 2011; Syroechkovskiy, 2006). Due to fast economic development, the number and size of natural wetlands have declined considerably in eastern China from the 1970s onwards (Gong et al., 2010; Niu et al., 2012). Despite the rapid decline of East Asian waterfowl species, critical knowledge gaps concerning their migration ecology remain. While the threatened swan goose (*Anser cygnoides*), breeding in Mongolia, has been satellite tracked (Batbayar et al., 2013), primary information regarding the migration routes and stopover patterns for other species, particularly those breeding in Siberia, is missing, severely limiting the efficiency of conservation actions.

As typical herbivorous waterfowl species, the Holarctic greater white‐fronted goose (*Anser albifrons*) and the Palearctic tundra bean goose (*Anser serrirostris*) breed in the Tundra and winter in the temperate zone (del Hoyo, Elliott, & Sargatal, 1992). Breeding populations in Russia have shown a rapid decline since the 1980s (Syroechkovskiy, 2006). East Asian populations mainly winter in eastern China, Korea, and Japan. The greater white‐fronted goose population wintering in the Yangtze River Floodplain in southeast China has shown a striking decline from 140,000 geese in 1987 to 18,000 in 2010, particularly in the provinces Jiangxi and Hunan (but not in Anhui Province) (Zhao, Cong, Barter, Fox, & Cao, 2012). The East Asian populations of tundra bean geese have steeply declined as well, with an 85%–90% decrease in the number of breeding birds compared to a century ago (Syroechkovskiy, 2006). The migration routes and stopover sites for the populations of these two goose species wintering in China are as yet unknown.

To fill the knowledge gap in the migration ecology of East Asian waterfowl, we use satellite tracking and spatial analyses to investigate spring migration routes, stopover sites, habitat selection, and site protection status of greater white‐fronted and tundra bean geese wintering in the Yangtze River Floodplain. We aim to (a) estimate the habitat utilization distribution of tracked geese and identify the stopover sites during spring migration; (b) summarize migration timing and distance and number of stopover sites and length of stay; (c) investigate habitat selection and site protection status along migration routes; and finally, (d) discuss the implications of our findings for the conservation of declining waterfowl species wintering in China.

## 2 MATERIALS AND METHODS

### 2.1 Goose capture and satellite transmitter deployment

**Figure 1:**
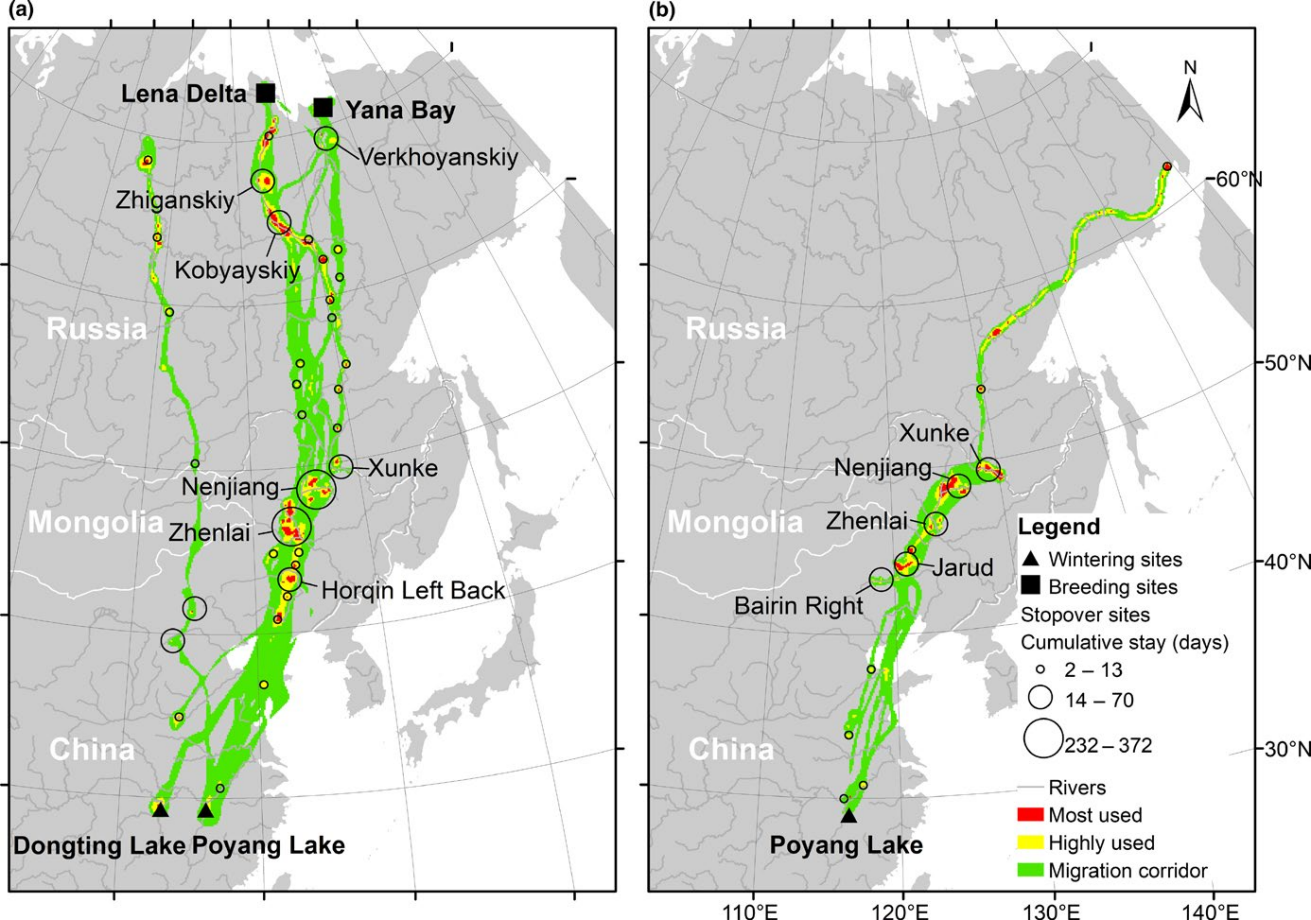
Locations of stopover sites plotted over the multibird level migration routes and utilization distribution of greater white‐fronted geese (*Anser albifrons* (a) based on 12 full tracks and six partial tracks) and tundra bean geese (*Anser serrirostris* (b) based on six partial tracks). The utilization distribution is represented by red, yellow, and green colors indicating 75%, 90%, and 99% cumulative probability contours calculated by the dynamic Brownian bridge movement model. Stopover sites were identified using the space–time permutation model from the SaTScan statistics. Stopover sites in China are named by county and those in Russia are named by district (only those main sites where at least two individuals stayed for cumulatively no less than 2 weeks are shown)

Approval for capture of and deploying transmitters on migratory birds was obtained from the Jiangxi Provincial Forestry Bureau (reference number: Ganlinban 201514 and 201570) and the Animal Ethics Committee at Tsinghua University (reference number: IACUC15‐SYL1). From 30 January to 2 February and from 7 to 16 December 2015, we captured 24 greater white‐fronted geese and 13 tundra bean geese with leg nooses and flat nets at Poyang Lake on the Yangtze River Floodplain, Jiangxi Province, China (29°N, 116°E; Figure 1). Birds were placed individually into bags and immediately transported to the closest handling station. We identified their sex (confirmed with molecular methods following (Fridolfsson & Ellegren, 1999)) and age (juvenile or adult) before equipping them with GPS‐GSM (Global Positioning System—Global System for Mobile Communications), solar‐powered loggers (See Supporting information Table S1 for a summary of logger information). Each logger was set to record GPS positions every 2 hr and send data back by SMS every 4–10 hr. The collected data used in this study included latitude and longitude (degree) and speed (km/hr). Birds were promptly released at the capture site after transmitters were deployed (the average time between capture and release was 6 hr).

### 2.2 Satellite tracking data

Of 37 birds, 24 spring migration tracks were collected, including 12 full spring migration tracks for nine greater white‐fronted geese in 2015 and 2016 (three individuals were tracked for 2 years), and 12 partial tracks for six greater white‐fronted geese and six tundra bean geese in 2016. Full tracks were defined as individuals that managed to return to the Yangtze River Plain the next season, whereas partial tracks were defined as individuals that travelled at least 1,500 km away from the wintering site before the signal was lost. We used the GPS locations covering the period from 3 days before birds left the wintering site until 3 days after they reached their likely breeding sites to describe a complete spring migration route (for partial tracks, data until the last available record were used). Detailed migration periods, count of days, and locations for each individual are reported in Supporting information Table S2. Despite missing GPS records due to satellite acquisition failure or low battery level, we collected on average ten locations per bird per day. All tracking data are stored in Movebank (https://www.movebank.org/) under ID 52997422, study “2015 Tsinghua waterfowl (Yangtze).”

### 2.3 Calculating utilization distribution, stopover sites, migration schedule, and travel distances

We used the dynamic Brownian bridge movement model from the “move” package (Kranstauber & Smolla, 2015) in the R Statistical software (http://www.r-project.org) to estimate the utilization distribution (the relative frequency of the use of a two‐dimensional area) with time series of tracking data (See R code in Supporting information Appendix S2). Cumulative probability contours were calculated to represent the relative use at both individual and multibird levels. The utilization distribution within the 75% contour was classified as the most intensively used areas where birds stopped over for extended periods of time, those between the 75% and 90% contours as highly used area with short flights, and those between the 90% and 99% contours as flight corridor with minimal stops. We define the core area as utilization distribution within 90% contours (both most used and highly used areas).

The space–time permutation model in SaTScan statistics (http://www.satscan.org) was applied to identify the locations and timing of space–time clusters that can be identified as stopover sites, that is, sites where birds spend extended periods of time during migration. For identified stopover sites, central locations and radiuses were used to describe the range of each site. The main stopover sites were defined as those where at least two individuals stayed for cumulatively no less than 2 weeks. Number of staying individuals and cumulative staying days were reported for these main stopover sites. Moreover, for each individual, we identified the departure and arrival dates, the flight distances in between stopover sites, and the duration of stays at each stopover site.

### 2.4 Analyzing habitat selection and site protection status

The 30 m global land cover product Finer Resolution Observation and Monitoring of Global Land Cover available from http://data.ess.tsinghua.edu.cn/ (Gong et al., 2013) was used to examine habitat use and selection by tracked geese at stopover sites. Eight land cover types were included as follows: water, croplands, forests, grasslands, wetlands, shrublands, barelands, and tundra. Manly’s habitat selection index (Manly, McDonald, Thomas, McDonald, & Erickson, 2002) was used to calculate the selection ratio of each land cover type during day and night in each main stopover site. Specifically, we divided the percentage of GPS locations on one land cover type by the percentage of pixels covered by this land cover type within the boundary of a specific stopover site (described by the center coordinates and radius of each site calculated by the space–time permutation model).

The boundary of protected areas (obtained from http://www.protectedplanet.net/) was used to investigate the protection status of the areas. We overlapped the protected areas with the GPS locations (location records in flight were excluded) of tracked geese and calculated the percentage of locations falling into protected areas. Using the identified multibird level utilization distribution, we calculated the percentage of the core areas that are under protection. Both habitat selection and protection status analyses were performed in the ArcGIS software (version 10.0, ESRI Inc., Redlands, CA, USA).

A detailed description on the estimation of the utilization distribution with the dynamic Brownian bridge movement model, the identification of stopover sites using the space–time permutation model, and the calculation of migration schedule, travel distances, number of stopover sites, and the length of stay can be found in Supporting information Appendix S1.

## 3 RESULTS

### 3.1 Migration routes, utilization distribution, and stopover site distribution

Spring migration corridors of the greater white‐fronted geese extended from the wintering sites along the Yangtze River Floodplain across the Northeast China Plain and east Mongolia to their likely breeding sites in Lena Delta and Yana Bay in northern Siberian lowland (Figure 1a). No full tracks were obtained for tundra bean geese and the farthest an individual reached was northeast Siberia (Figure 1b). All tracked individuals stopped over in the Northeast China Plain. Stopover areas were concentrated in the Northeast China Plain between 40‐ and 50‐degree latitude (for both species), and the Lena River Basin, above a latitude of 60 degrees (for the greater white‐fronted geese).

A total of seven and five main stopover sites (space–time clusters with *p* values <0.05, sites used by at least two individuals for no less than cumulatively 2 weeks) were identified based on 18 tracks of 15 individual greater white‐fronted geese and six tracks of six individual bean geese during spring migration (Figure 1). Detailed information about the central location and radius of stopover sites, cumulative staging periods, and the number of individuals that used each site are summarized in Supporting information Table S3. The locations of the main stopover sites showed a good match with the intensively used areas at the multibird level. Four of seven main stopover sites of greater white‐fronted geese were located in the Northeast China Plain, including Horqin Left Back in Inner Mongolia, Zhenlai County at the boundary of Jilin Province and Inner Mongolia, Nenjiang, and Xunke County in Heilongjiang Province. Zhenlai and Nenjiang are the two most frequently utilized sites where geese spent cumulatively 372 days (14 individuals) and 232 days (11 individuals; Supporting information Table S3). Additionally, main stopover sites were found in Russia (Kobyayskiy, Zhiganskiy, and Verkhoyanskiy Districts), where geese stayed cumulatively 18 (two individuals) to 32 (six individuals) days before they migrated to their likely breeding site at the Lena Delta and the Yana Delta (Supporting information Table S3). Based on six partial tracks of six individuals, all five main stopover sites for tundra bean geese were located in the Northeast China Plain, and three of these sites (Zhenlai, Nenjiang, and Xunke County) overlapped with the main stopover sites used by greater white‐fronted geese.

### 3.2 Migration schedule, distances, and stopover patterns

Most greater white‐fronted geese (12 of 15 birds) departed from the Yangtze River Floodplain at the end of March, stopped over at the Northeast China Plain and Russia, and arrived at their breeding site in northeast Siberian lowland between the end of May and the beginning of July (Table 1, Figure 1). Based on full tracks only, the period of spring migration lasted 53–92 days, stretching over 5,000 km, with a total duration of stay of 43–81 days between 4 and 15 stopover sites, of which 32–49 days were spent in the Northeast China Plain. Tundra bean geese in general departed 1 month earlier (at the end of February or the beginning of March) from Yangtze. All tundra bean geese stopped over at the Northeast China Plain and one individual reached Severo‐Evenskiy in Russia, 5,709 km away from Yangtze, before the signal was lost. Geese with full tracks spent their longest staging period in the Northeast China Plain (Table 1, Figure 1).

**Table 1:** Summary of the spring migration schedule, travel distances, number of stopover sites, and duration of stay of 15 greater white‐fronted geese (*Anser albifrons*) during 2015 and 2016 and six tundra bean geese (*Anser serrirostris*) in 2016

ID	Age	Sex	Track	Depart YRF	Reach NSL/Last record	Migration period (day)	Distance (km)	Stopover sites (*n*)	Cumulative duration (day)	Stopover NCP (day)
E001(GWFG2015)	A	M	Full	28‐Mar	29‐May	62	5,027	3	43	40
E002(GWFG2015)	A	F	Full	31‐Mar	5‐Jun	66	5,281	10	52	33
E003(GWFG2015)	J^a^	F	Full	26‐Mar	11‐Jun	77	5,328	2	52	49
E005(GWFG2015)	A	M	Full	22‐Mar	5‐Jun	75	5,340	5	63	46
E002(GWFG2016)	A	F	Full	25‐Mar	31‐May	67	5,279	6	49	41
E003(GWFG2016)	A	F	Full	26‐Mar	2‐Jun	68	5,188	3	52	47
E005(GWFG2016)	A	M	Full	25‐Mar	28‐May	64	5,002	4	55	49
E010(GWFG2016)	A	M	Full	10‐Apr	11‐Jul	92	5,563	12	81	32
E013(GWFG2016)	A	M	Full	1‐Apr	17‐Jun	77	5,462	6	66	43
E018(GWFG2016)	A	M	Full	27‐Mar	21‐May	55	4,964	3	48	39
E022(GWFG2016)	A	M	Full	27‐Mar	19‐May	53	5,314	6	50	38
H021(GWFG2016)	A	M	Full	17‐Mar	20‐May	64	5,096	6	54	38
E017(GWFG2016)	A	F	Partial	25‐Mar	8‐May	44	2,825	3	38	38
H003(GWFG2016)	A	M	Partial	26‐Mar	10‐May	45	2,201	3	42	40
H005(GWFG2016)	A	F	Partial	26‐Mar	9‐May	44	2,406	3	40	38
H006(GWFG2016)	J	F	Partial	28‐Mar	9‐May	42	2,533	3	32	32
H016(GWFG2016)	A	F	Partial	25‐Mar	19‐May	55	2,541	3	48	48
H018(GWFG2016)	A	M	Partial	18‐Apr	17‐May	29	2,305	3	22	22
E007(TUBG2016)	A	F	Partial	28‐Feb	11‐May	73	5,709	8	61	54
E024(TUBG2016)	A	F	Partial	6‐Mar	9‐Apr	34	2,922	4	22	18
H004(TUBG2016)	A	F	Partial	1‐Mar	1‐May	61	2,899	4	54	54
H019(TUBG2016)	A	F	Partial	1‐Mar	26‐Mar	25	1,756	1	22	22
H022(TUBG2016)	A	M	Partial	26‐Feb	3‐Mar	6	1,508	2	4	2
B012(TUBG2016)	A	M	Partial	5‐Mar	27‐Apr	53	2,446	3	45	45

Notes

ID: E, ecotone telemetry; GWFG, greater white‐fronted goose; H, Hunan Global Messenger Technology Co. Ltd; B, Blueoceanix Technology Co. Ltd; TUBG, tundra bean goose; Age: A, adult; J, juvenile; Sex: F, female; M, male; NSL, northeast Siberian lowland; NCP, Northeast China Plain; YRF, Yangtze River Floodplain.

a A juvenile when captured in 2015 and an adult in 2016.

### 3.3 Habitat selection and protection status in stopover sites

During both day and night time, the selection rate for water was highest compared to other land cover types for both greater white‐fronted geese and tundra bean geese at most stopover sites (Table 2). Specifically, during the day, both species show a preference for cropland, with tundra bean geese showing a slightly higher preference than greater white‐fronted geese. Both species show some preference for other foraging lands such as grass, wetland, and bareland in a few sites.

**Table 2:** Standardized selection rate for the greater white‐fronted geese (*Anser albifrons*) and tundra bean geese (*Anser serrirostris*) on different land cover types during day (top) and night (bottom) at main stopover sites during spring migration

	Greater white‐fronted geese							Tundra bean geese				
	Horqin Left Back	Zhen‐lai	Nen‐jiang	Xun‐ke	Kobyay‐skiy	Zhig‐anskiy	Verkho‐yanskiy	Bairin Right	Jarud	Zhen‐lai	Nen‐jiang	Xun‐ke
Cumulative days	56	372	232	20	32	23	18	26	27	17	70	36
Crop	22	9	7	12	–	–	–	100	6	3	100	63
Forest	0	0	0	0	4	5	4	0	17	0	3	0
Grass	7	15	10	0	24	6	1	0	3	14	55	1
Shrub	0	12	0	0	0	0	0	24	3	0	0	0
Wetland	0	8	48	0	0	0	0	0	0	100	0	0
Water	100	100	100	100	100	30	100	92	100	37	99	100
Tundra	–	–	–	–	0	0	68	–	–	–	–	–
Bareland	4	37	7	9	5	100	37	19	4	12	83	21
Cumulative nights	55	371	232	20	26	11	0^a^	27	27	18	70	36
Crop	0	3	1	3	–	–	–	2	1	0	3	5
Forest	0	0	0	0	3	1	–	0	1	0	0	0
Grass	0	10	8	0	27	18	–	0	1	1	17	1
Shrub	0	0	0	0	0	0	–	0	1	0	0	0
Wetland	0	0	0	0	0	0	–	0	0	0	0	0
Water	100	100	100	100	100	30	–	100	100	100	100	100
Tundra	–	–	–	–	0	0	–	–	–	–	–	–
Bareland	0	12	1	1	0	100	–	3	1	7	1	7

Selection rates were standardized to 0–100 to assist comparison.

a No night habitat use as this site is in polar day.

**Figure 2:**
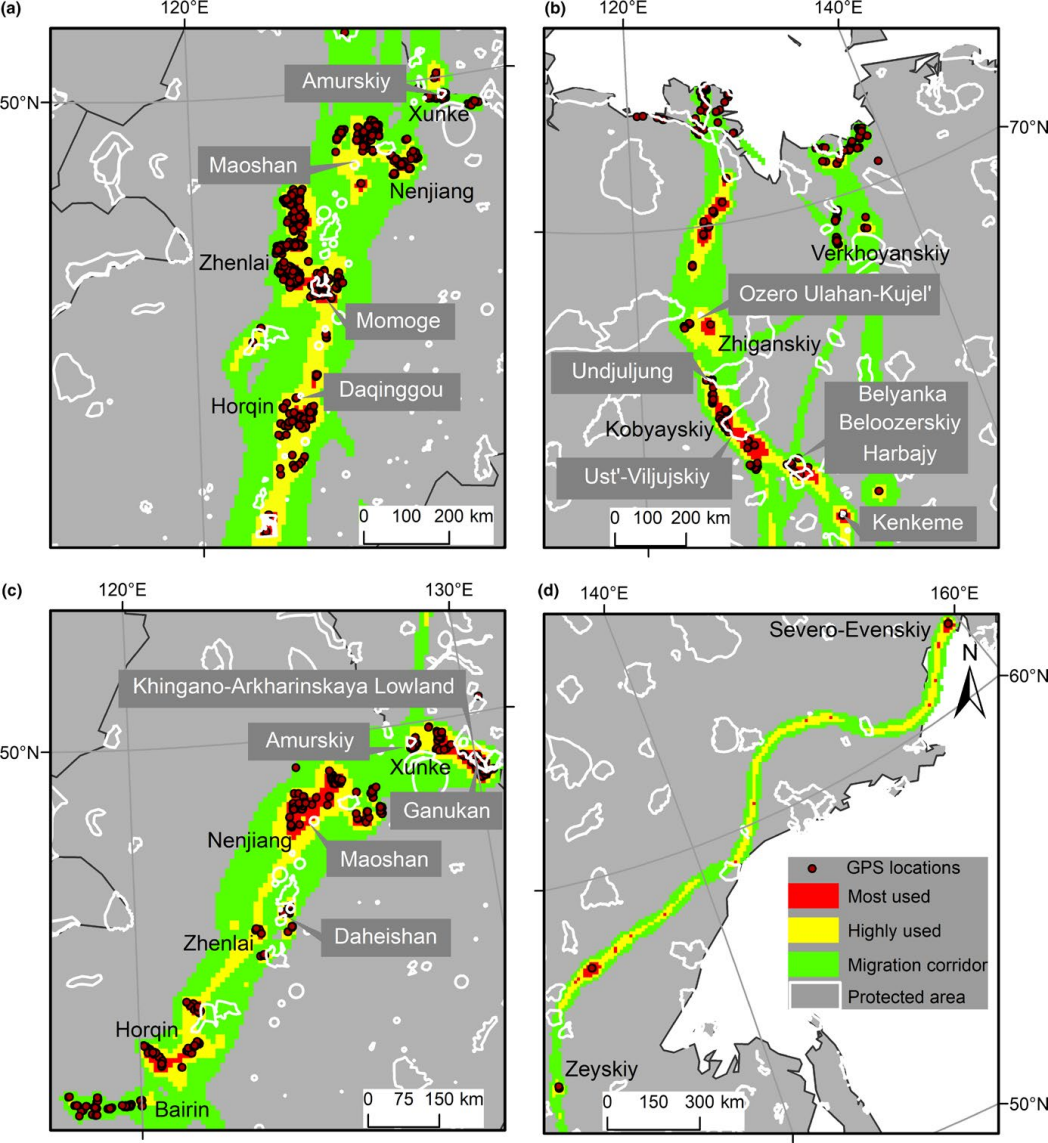
Overlap of protected areas and multibird level utilization distribution of tracked greater white‐fronted geese (*Anser albifrons* [a] and [b]) and tundra bean geese (*Anser serrirostris* [c] and [d]). Brown dots indicate recorded GPS locations. The utilization distribution is represented by red, yellow, and green colors indicating 75%, 90%, and 99% cumulative probability contours calculated by the dynamic Brownian bridge movement model

In terms of site protection status, a total of 86% (9,100 of 10,572, greater white‐fronted geese) and 85% (2,155 of 2,474, tundra bean geese) of GPS locations were recorded outside of protected areas. When comparing the distribution of the utilization distribution of geese with the protected areas, 94% (206,269 of 219,100 km^2^) of core areas (including most and highly used areas) did not overlap with the protected areas for greater white‐fronted geese and 92% (104,388 of 113,300 km^2^) for tundra bean geese. The protected areas available for geese in China are less than in Russia (Figure 2). Details on the designation and protection status of these overlapping areas are summarized in Supporting information Table S4.

## 4 DISCUSSION

We observed a “long‐stay and short‐travel” spring migration strategy for East Asian greater white‐fronted geese and tundra bean geese. Both species tend to spend an extended period of time in a few neighboring stopover sites at the Northeast China Plain. A “long‐stay and short‐travel” strategy has also been documented for mallards (*Anas platyrhynchos*) wintering in Japan during the spring migration (Yamaguchi et al., 2008) and Siberian crane (*Grus leucogeranus*) during the autumn migration from northeastern Siberia to China (Kanai et al., 2002). A long‐stay in a few stopover sites may facilitate replenishing energy reserves, but may also reflect a limited number of suitable stopover sites along the migration route.

The Northeast China Plain is found to be a critical midway stopover area for greater white‐fronted geese and tundra bean geese wintering in China. Individuals with full tracks spent most of their staging time (over a month) in this area. Besides geese, Endangered Siberian cranes also use this region extensively during spring (Kanai et al., 2002). Despite its importance for migratory waterbirds, the Northeast China Plain experienced a considerable loss of natural waterbodies and wetlands in the period from 1990 to 2000 (Gong et al., 2010), with inland marshes in particular being converted into other land use types (Niu et al., 2012). The deterioration of the crucial stopover sites in the Northeast China Plain, together with the natural habitat loss in the wintering sites (Yu et al., 2017), is probably a main reason for the dramatic decline of waterfowl wintering in China. There are regular waterfowl surveys along the Yangtze River Floodplain. However, no census data are available to evaluate the situation of migratory waterfowl at the Northeast China Plain. Future ground surveys should be carried out to validate the habitat use of migratory waterfowl at a population level in these core stopover areas.

By further investigating the habitat selection of the two study species at their main stopover sites, we show that water yields the highest selection rate (area used against area available at a specific stopover site) across most sites, followed by potential foraging lands including crop, wetland, bareland, and grass. Waterbodies are typical roosting sites and geese prefer foraging sites in close proximity to their roosts (Ackerman et al., 2006; Elphick, 2008; Ely, 1992; Moriguchi, Amano, & Ushiyama, 2013; Rosin et al., 2012; Si et al., 2011; Zhang, Li, Yu, & Si, 2018). Geese stopping over in the Northeast China Plain were observed to graze on newly flushed spring meadows near waterbodies after ice melt (personal communication with local farmers). Grass not being highly selected might reflect misclassification of short grass into water, wetland, or bareland, as the young meadows geese prefer often occur near water (Aharon‐Rotman et al., 2017). White‐fronted geese and tundra bean geese staging in Central Europe and Japan utilized agricultural fields in close proximity to water (Jankowiak et al., 2015; Rosin et al., 2012; Shimada, 1997). Waterbodies are most vulnerable to the land conversion associated with the development of agriculture. Partial conversion of natural wetlands into agriculture could be beneficial for foraging, as exemplified by the use of cropland by our tracked geese. However, as water was most highly selected by these birds, completely eradicating waterbodies would be disastrous to waterfowl. Hence, the protection of waterbodies used as roosting area, especially those surrounded by foraging land, is critical for waterfowl survival.

Besides water and natural foraging lands, cropland was highly selected at some sites. Grains generally contain more energy than grasses (Shimada, 2002), and since 1950s Arctic‐breeding geese wintering in Europe and America have shifted their foraging to agricultural land including cornfields, winter wheat, rice fields, and pastures, to benefit from the highly profitable resources there (Ackerman et al., 2006; Fox, Elmberg, Tombre, & Hessel, 2017; Fox et al., 2005; Krapu, Reinecke, Jorde, & Simpson, 1995; Lane, Azuma, & Higuchi, 1998; Moriguchi et al., 2013; Rosin et al., 2012; Si et al., 2011). Geese stopping over in the Northeast China Plain prefers aggregated waterbodies, surrounded by scattered croplands at the foraging scale (Zhang et al., 2018). Moreover, maize harvested by combine harvesters in this area also increases food availability (due to more leftover kernels being available than under manual harvesting) for spring‐staging geese (personal communication with local farmers). Agricultural lands are therefore heavily used by geese, especially when lakes are still frozen upon arrival.

Increased foraging opportunities related to agriculture development have caused rapid goose population expansion in Europe and North America (Abraham, Jefferies, & Alisauskas, 2005; Van Eerden, Zijlstra, van Roomen, & Timmerman, 1996). However, massive wetland loss, in particular due to the conversion of important roosting lakes and wetlands into agricultural land, is expected to negatively affect waterfowl survival. Moreover, intensive human activities can reduce foraging effectiveness. Geese wintering along the Yangtze are confined to natural habitats and avoid the surrounding farmlands (Aharon‐Rotman et al., 2017; Yu et al., 2017). Migratory geese in their wintering and staging sites in China particularly select areas with a low level of human disturbance (Li, Si, Ji, & Gong, 2017; Zhang et al., 2018). In order to generate concrete protection measures in the Northeast China Plain, future studies need to further investigate these potential sources of bird–human conflict.

Most areas utilized during spring migration by our tracked greater white‐fronted and tundra bean geese are located outside of the protected area network. A similar scenario was found for Siberian crane (Kanai et al., 2002). A lack of protected areas along flyways is a general problem for migratory bird species all around the world (Runge et al., 2015). Geese are highly adaptable and able to survive under simple habitat conservation and restoration measures (e.g. preserve/restore roosting areas and allow grazing on agricultural land). Based on the core utilized area reported in this study and with information provided by future ground surveys, the network of protected areas of East Asian waterfowl could be improved by including core roosting areas in critical spring‐staging sites along the migratory flyway.

## 5 CONCLUSIONS

We analyzed the spring migration pattern of East Asian greater white‐fronted geese and tundra bean geese wintering in China for the first time. Crucially, the Northeast China Plain (covering Heilongjiang, Inner Mongolia, and Jilin Provinces) is found to support the most intensively used spring stopover sites for greater white‐fronted geese and (albeit based on partial tracks) tundra bean geese. The protection of waterbodies used as the core roosting area, especially those surrounded by foraging land, is critical for the survival of waterfowl. As both tracked species partially forage on agricultural land in the Northeast China Plain, potential bird–human conflict needs to be further studied. Moreover, hardly any of the areas that geese intensively utilize during their spring migration are formally protected. We recommend integrating waterbodies at critical stopover sites, used as core roosting area, into the network of protected areas. Field surveys should target these key stopover sites to further validate their importance and generate site‐specific conservation measures to protect declining waterfowl wintering in China. Although only a limited number of individuals were tracked (including some partial tracks that do not cover the entire spring migration route) and bearing loggers might affect migration behavior, this study provides the first insight into important stopover sites for East Asian waterfowl wintering in China. Our study illustrates how satellite tracking combined with spatial analyses can be used to guide conservation efforts.

## CONFLICT OF INTEREST

None declared.

## AUTHOR CONTRIBUTION

Y.S., Y.X., F.X., W.Z., B.W., and P.G. conceived and designed the experiments; Y.S., Y.X., F.X., W.Z., B.W., J.W., G.L., H.L., J.T., S.B., and T.Z. performed the experiments; Y.S., Y.X., F.X., X.L., W.Z., and J.W. analyzed the data; G.L., H.L., J.T., S.B., T.Z., W.F.D.B., and H.H.T.P contributed materials and analysis tools; Y.S., Y.X., F.X., X.L., W.Z., B.W., J.W., J.T., W.F.G.B., H.H.T.P., and P.G. wrote the manuscript. All authors edited the manuscript and approved the final version for publication.
