# Supplementary material for: Spring migration patterns, habitat use, and stopover site protection status for two declining waterfowl species wintering in China as revealed by satellite tracking
Source: Ecol Evol. 2018 May 24;8(12):6280–9. doi: 10.1002/ece3.4174 (PMC6024133; doi:10.1002/ece3.4174)
Supplement: Supplementary file 1 [file ECE3-8-6280-s001.docx]

Table S1. Summary of three GPS-GSM (Global Positioning System - Global System for Mobile Communications) logger types.

| Logger type  (number of individuals) | Logger weight (g) | Tag type | Logger percentage body weight | Location error (m) | Company |
| --- | --- | --- | --- | --- | --- |
| IBIS series neck loggers (20) | 22 | Neckband | 1% | 20 | Ecotone Telemetry, Gdynia, Poland |
| HQNN series neck loggers (15) | 26 | Neckband | 1% | 0-5, 10-30, >100 | Hunan Global Messenger Technology Co. Ltd, Xiangtan, China |
| ANIT series back loggers (2) | <25 | Backpack | 1% | 5, 10, 20, 100 | Blueoceanix Technology Co. Ltd, Tianjin, China |
