# Supplementary material for: Spring migration patterns, habitat use, and stopover site protection status for two declining waterfowl species wintering in China as revealed by satellite tracking
Source: Ecol Evol. 2018 May 24;8(12):6280–9. doi: 10.1002/ece3.4174 (PMC6024133; doi:10.1002/ece3.4174)
Supplement: Supplementary file 2 [file ECE3-8-6280-s002.docx]

Table S2 Summary of GPS records obtained for each individual greater white-fronted goose (*Anser albifrons*) and tundra bean goose (*Anser serrirostris*) during spring migration in 2015 and 2016. Individuals travel at least 1500 km away from the wintering site were included in partial tracks.

| Bird ID | Track | 3 days before departure | 3 days after arrival/last record | Count of Days (n) | Count of locations (n) |
| --- | --- | --- | --- | --- | --- |
| E001(GWFG2015) | Full | 2015/3/25 | 2015/5/29 | 57 | 505 |
| E002(GWFG2015) | Full | 2015/3/28 | 2015/6/8 | 73 | 693 |
| E003(GWFG2015) | Full | 2015/3/23 | 2015/6/14 | 84 | 712 |
| E005(GWFG2015) | Full | 2015/3/19 | 2015/6/8 | 82 | 719 |
| E002(GWFG2016) | Full | 2016/3/22 | 2016/6/3 | 73 | 561 |
| E003(GWFG2016) | Full | 2016/3/23 | 2016/6/5 | 75 | 688 |
| E005(GWFG2016) | Full | 2016/3/22 | 2016/5/31 | 70 | 801 |
| E010(GWFG2016) | Full | 2016/4/7 | 2016/7/14 | 99 | 873 |
| E013(GWFG2016) | Full | 2016/3/29 | 2016/6/20 | 84 | 919 |
| E018(GWFG2016) | Full | 2016/3/24 | 2016/5/24 | 62 | 620 |
| E022(GWFG2016) | Full | 2016/3/24 | 2016/5/24 | 62 | 529 |
| H021(GWFG2016) | Full | 2016/3/14 | 2016/5/23 | 70 | 789 |
| E017(GWFG2016) | Partial | 2016/3/22 | 2016/5/8 | 48 | 531 |
| H003(GWFG2016) | Partial | 2016/3/23 | 2016/5/10 | 49 | 551 |
| H005(GWFG2016) | Partial | 2016/3/23 | 2016/5/9 | 46 | 434 |
| H006(GWFG2016) | Partial | 2016/3/25 | 2016/5/9 | 44 | 456 |
| H016(GWFG2016) | Partial | 2016/3/22 | 2016/5/19 | 58 | 515 |
| H018(GWFG2016) | Partial | 2016/4/15 | 2016/5/17 | 31 | 309 |
| E007(TUBG2016) | Partial | 2016/2/25 | 2016/5/11 | 77 | 878 |
| E024(TUBG2016) | Partial | 2016/3/3 | 2016/4/9 | 37 | 392 |
| H004(TUBG2016) | Partial | 2016/2/28 | 2016/5/1 | 55 | 468 |
| H019(TUBG2016) | Partial | 2016/2/28 | 2016/3/26 | 28 | 240 |
| H022(TUBG2016) | Partial | 2016/2/23 | 2016/3/3 | 10 | 99 |
| B012(TUBG2016) | Partial | 2016/3/2 | 2016/4/27 | 57 | 591 |
| Total | - | - | - | 1431 | 13873 |

ID: E = Ecotone Telemetry, H = Hunan Global Messenger Technology Co. Ltd, B = Blueoceanix Technology Co. Ltd; GWFG = greater white-fronted goose, TUBG = tundra bean goose.
