# Supplementary material for: Spring migration patterns, habitat use, and stopover site protection status for two declining waterfowl species wintering in China as revealed by satellite tracking
Source: Ecol Evol. 2018 May 24;8(12):6280–9. doi: 10.1002/ece3.4174 (PMC6024133; doi:10.1002/ece3.4174)
Supplement: Supplementary file 3 [file ECE3-8-6280-s003.docx]

Table S3 Location of stopover sites, cumulative bird staging periods and the number of individuals used each site for 15 grater-white fronted geese (*Anser albifrons*) and six tundra bean geese (*Anser serrirostris*) in 2015 and 2016. Main stopover sites (used by at least two individuals for no less than cumulatively two weeks) are marked in bold.

| No. | Species | Center location (Lat) | Center Location (Lon) | Radius (km) | County/District | Province | cumulative period (day) | Individual (n) |
| --- | --- | --- | --- | --- | --- | --- | --- | --- |
| 1 | GWFG | 30.60601 | 117.0779 | 12.36 | Huaining | Anhui | 12 | 3 |
| 2 | GWFG | 34.93882 | 114.0207 | 1.19 | Yanjin | Henan | 12 | 1 |
| 3 | GWFG | 36.89423 | 120.4009 | 11.65 | Laixi | Shandong | 2 | 1 |
| 4 | GWFG | 39.57542 | 113.3428 | 4.62 | Ying | Shanxi | 17 | 1 |
| 5 | GWFG | 40.8344 | 121.7112 | 42.41 | Panshan | Liaoning | 5 | 1 |
| 6 | GWFG | 41.58642 | 115.0053 | 35.31 | Zhangbei | Hebei | 26 | 1 |
| 7 | GWFG | 42.19503 | 122.6184 | 36.04 | Xinmin | Liaoning | 3 | 1 |
| **8** | **GWFG** | **43.20957** | **122.8901** | **47.37** | **Horqin Left Back** | **Inner Mongolia** | **56** | **8** |
| 9 | GWFG | 44.07122 | 123.4678 | 4.29 | Shuangliao | Jilin | 2 | 1 |
| 10 | GWFG | 44.82833 | 123.842 | 9.64 | Qian'an | Jilin | 2 | 1 |
| 11 | GWFG | 44.8428 | 121.6537 | 39.7 | Horqin Right Middle | Inner Mongolia | 5 | 2 |
| **12** | **GWFG** | **46.42174** | **123.4029** | **158.2** | **Zhenlai** | **Jilin** | **372** | **14*** |
| **13** | **GWFG** | **48.51568** | **125.9244** | **115.48** | **Nenjiang** | **Heilongjiang** | **232** | **11*** |
| **14** | **GWFG** | **49.68699** | **128.4693** | **74.29** | **Xunke** | **Heilongjiang** | **20** | **2** |
| 15 | GWFG | 50.41958 | 114.7152 | 0.98 | Ononskiy | Chita | 3 | 1 |
| 16 | GWFG | 52.03052 | 128.7242 | 2.18 | Mazanovskiy | Armur | 8 | 1 |
| 17 | GWFG | 53.11995 | 125.407 | 20.6 | Tahe | Heilongjiang | 4 | 2 |
| 18 | GWFG | 54.3323 | 129.4776 | 1.11 | Zeyskiy | Armur | 3 | 1 |
| 19 | GWFG | 54.99608 | 125.2286 | 0.15 | Tyndinskiy | Armur | 2 | 1 |
| 20 | GWFG | 55.74048 | 130.7731 | 0.41 | Neryungri-W | Sakha | 5 | 1 |
| 21 | GWFG | 56.22463 | 125.8855 | 3.57 | Neryungri-E | Sakha | 2 | 1 |
| 22 | GWFG | 58.64408 | 130.2257 | 0.15 | Aldanskiy | Sakha | 2 | 1 |
| 23 | GWFG | 59.53978 | 111.0667 | 25.62 | Lenskiy | Sakha | 3 | 1 |
| 24 | GWFG | 59.7385 | 130.4324 | 40.56 | Oroqin | Inner Mongolia | 2 | 1 |
| 25 | GWFG | 59.7385 | 130.4324 | 40.56 | Amginskiy | Sakha | 5 | 1 |
| 26 | GWFG | 60.94342 | 132.2013 | 0.36 | Ust'-Mayskiy | Sakha | 2 | 1 |
| 27 | GWFG | 62.2134 | 130.6254 | 1.65 | Magino-Kangalasskiy | Sakha | 7 | 1 |
| 28 | GWFG | 62.59377 | 132.8221 | 0.37 | Tattinskiy | Sakha | 2 | 1 |
| 29 | GWFG | 63.56438 | 129.2702 | 36.6 | Namskiy | Sakha | 10 | 2 |
| 30 | GWFG | 63.97058 | 108.4148 | 43.94 | Mirninskiy | Sakha | 9 | 1 |
| **31** | **GWFG** | **64.88591** | **125.6033** | **113.22** | **Kobyayskiy** | **Sakha** | **32** | **6*** |
| **32** | **GWFG** | **67.50578** | **123.9286** | **5.81** | **Zhiganskiy** | **Sakha** | **23** | **5** |
| 33 | GWFG | 68.51238 | 105.1691 | 31.64 | Ilimpiyskiy | Everk | 8 | 1 |
| **34** | **GWFG** | **69.23296** | **135.5658** | **78.65** | **Verkhoyanskiy** | **Sakha** | **18** | **2*** |
| 35 | GWFG | 70.15461 | 125.9577 | 52.8 | Bulunskiy | Sakha | 13 | 4* |
| 1 | TUBG | 29.97782 | 115.9836 | 13.39 | Huangmei | Hubei | 2 | 1 |
| 2 | TUBG | 30.78663 | 117.3383 | 0.64 | Guichi | Anhui | 2 | 1 |
| 3 | TUBG | 33.86147 | 116.2905 | 4.23 | Yongcheng | Henan | 9 | 1 |
| 4 | TUBG | 37.87483 | 117.9945 | 1.73 | Wudi | Shandong | 2 | 1 |
| **5** | **TUBG** | **43.34458** | **118.9061** | **90.5** | **Bairin Right** | **Inner Mongolia** | **27** | **2** |
| **6** | **TUBG** | **44.24853** | **121.0656** | **77.9** | **Jarud** | **Inner Mongolia** | **27** | **3** |
| 7 | TUBG | 45.08732 | 121.5856 | 24.77 | Horqin Right Middle | Inner Mongolia | 5 | 1 |
| **8** | **TUBG** | **46.54924** | **123.8482** | **54.19** | **Zhenlai** | **Jilin** | **18** | **3** |
| **9** | **TUBG** | **48.70838** | **126.2665** | **141.58** | **Nenjiang** | **Heilongjiang** | **70** | **4** |
| **10** | **TUBG** | **49.44172** | **129.1626** | **86.64** | **Xunke** | **Heilongjiang** | **36** | **2** |
| 11 | TUBG | 54.3004 | 129.8033 | 6.51 | Zeyskiy | Amur | 2 | 1 |
| 12 | TUBG | 61.98495 | 159.3467 | 2.46 | Severo-Evenskiy | Maga Buryatdan | 2 | 1 |

GWFG = greater white-fronted goose, TUBG = tundra bean goose; *some individuals used the specific site in both 2015 and 2016 spring.
