# Supplementary material for: Spring migration patterns, habitat use, and stopover site protection status for two declining waterfowl species wintering in China as revealed by satellite tracking
Source: Ecol Evol. 2018 May 24;8(12):6280–9. doi: 10.1002/ece3.4174 (PMC6024133; doi:10.1002/ece3.4174)
Supplement: Supplementary file 4 [file ECE3-8-6280-s004.docx]

Table S4 The designation and protected status of protected areas overlapped with the core areas for greater white-fronted geese (*Anser albifrons*) and tundra bean geese (*Anser serrirostris*).

| Name | Species | Designation | Designation  type | Country | Status^*^ | Designated year |
| --- | --- | --- | --- | --- | --- | --- |
| Daqinggou | GWFG | Nature Reserve | National | China | Designated | 1988 |
| Momoge | GWFG | Nature Reserve | National | China | Designated | 1981 |
| Maoshan | GWFG&TUBG | Nature Reserve | National | China | Designated | 1988 |
| Daheishan | TUBG | Nature Reserve | National | China | Designated | 1986 |
| Amurskiy | GWFG&TUBG | Zakaznik | National | Russia | Designated | 1967 |
| Kenkeme | GWFG | Resource Reserve | National | Russia | Designated | 1996 |
| Belyanka | GWFG | Zakaznik | National | Russia | Designated | - |
| Beloozerskiy | GWFG | Zakaznik | National | Russia | Designated | 1974 |
| Harbajy | GWFG | Resource Reserve | National | Russia | Designated | 1996 |
| Ust'-Viljujskiy | GWFG | Zakaznik | National | Russia | Designated | 1986 |
| Undjuljung | GWFG | Zakaznik | National | Russia | Designated | 1994 |
| Ozero Ulahan-Kujel' | GWFG | Protected Landscape | National | Russia | Designated | 1994 |
| Khingano-Arkharinskaya Lowland | TUBG | Ramsar site | International | Russia | Designated | 1994 |
| Ganukan | TUBG | Zakaznik | National | Russia | Designated | 1985 |

* Designated indicates the protected status is recognized or dedicated through legal means, which implies specific binding commitment to conservation in the long term. GWFG = greater white-fronted goose, TUBG = tundra bean goose.
