# Supplementary material for: Spring migration patterns, habitat use, and stopover site protection status for two declining waterfowl species wintering in China as revealed by satellite tracking
Source: Ecol Evol. 2018 May 24;8(12):6280–9. doi: 10.1002/ece3.4174 (PMC6024133; doi:10.1002/ece3.4174)
Supplement: Supplementary file 5 [file ECE3-8-6280-s005.docx]

Detailed description on the methods

1. Estimating utilization distribution with the dynamic Brownian bridge movement model

The Brownian Bridge Movement Model (BBMM) ([Horne et al. 2007](#_ENREF_4)) is widely adopted to approximate animal utilization distributions (the relative frequency of the use of a two-dimensional area) with time series of tracking data. The BBMM is parameterized based on the time and distance between consecutive locations, the distribution of telemetry location errors, and the Brownian motion variance $\sigma_{m}^{2}$. Based on the BBMM, the dynamic Brownian Bridge Movement Model ([Kranstauber et al. 2012](#_ENREF_6)) improves the estimation of animal movements by identifying changes in speed and directions, calculating separate $\sigma_{m}^{2}$ values for different subsets of locations that correspond to different behaviours (e.g., foraging or travelling). The dynamic Brownian Bridge Movement Model determines whether there is a behavioral change along the animal’s movement path by comparing the likelihood statistics. Specifically, within a moving window covering a certain number of locations, the Bayesian information Criterion of the model with one $\sigma_{m}^{2}$ estimation is compared to that of the model with two estimates of $\sigma_{m}^{2}$. The model with a lower Bayesian information Criterion value is selected. The $\sigma_{m}^{2}$ is estimated by a leave-one-out method, and a margin size (*m*) with a minimum of three locations (*w*) is required for the calculation. Enlarging *w* improves the reliability of $\sigma_{m}^{2}$ but increases the chance of missing short changes in behavior, whereas enlarging *m* enhances the power to detect behavioral changes at the risk of not detecting breakpoints within the margin.

A window size of 31 locations with a margin size of 11 locations was selected based on the visual inspection of the tracking data ([Kranstauber et al. 2012](#_ENREF_6)). We used utilization distributions at a 10*10 km resolution to calculate the relative occupation time of each goose along the spring migration route. A multi-bird level utilization distribution for each species was generated by weighing each individual-bird utilization distribution with its migration duration. The cumulative utilization distribution sum was then re-scaled to one to estimate the multi-bird utilization distribution along the spring migration route ([Palm et al. 2015](#_ENREF_9)).

1. Identifying stopover sites using the space-time permutation model

The space-time permutation model ([Kulldorff et al. 2005](#_ENREF_8)) is defined by a cylindrical window with a circular base representing space and height representing time. The cylinder starts with a single point and increases its base and height until it reaches a maximum value. The maximum value for the base and height is selected based on the migration behavior of targeted species. In our case the maximum radius for the base was an empirical estimation of the longest distance birds might travel during their stay at the stopover sites, and for the height this was the potential length of stay at these sites. We calculated the expected number of GPS locations based on complete spatial randomness, and measured a Poisson generalized likelihood ratio using the expected and observed number of GPS locations to determine whether the cylinder contained a cluster or not ([Kulldorff et al. 2005](#_ENREF_8)). The significant level of the identified clusters was evaluated by 999 Mont Carlo simulation, through comparing the rank of the maximum likelihood from the observed dataset with the random dataset.

For the best performance, the maximum spatial scanning window should not exceed 50% of all GPS locations, and the temporal window should be set to no more than 50% of the study period ([Kulldorff 2006](#_ENREF_7)). We thereby chose 50 km as the maximal spatial scanning window as the maximum foraging flight distance for American and European geese are generally smaller than this ([Ackerman et al. 2006](#_ENREF_1); [Johnson et al. 2014](#_ENREF_5); [Si et al. 2011](#_ENREF_10)). Considering birds need to stay in stopover sites for at least 48 hour to settle and refuel ([Drent et al. 2006](#_ENREF_3)), a minimum temporal scanning window of two days and a maximum temporal window covering 50% of the spring migration period were selected. GPS locations record in flight (with a speed > 1 km/hour) were excluded from the analysis.

1. Calculating migration schedule, travel distances, number of stopover sites and the length of stay

The date that an individual bird started spring migration was defined as the first day it was known to have left the wintering location and continued to be absent from that area ([Bustnes et al. 2010](#_ENREF_2)). The date of arriving at the breeding sites was defined as the first day an individual arrived at the most northern site and a continuous stay was observed. The duration of the stay at each stopover site was defined as the number of days between the first date a bird appeared at a specific site and the last date it was recorded at that stopover site. The total number of migration days for each tracked goose was defined as the period between the date a bird left the wintering site and the first day it arrived at the breeding site (full tracks) or till the last day it was recorded (partial tracks).The travel distance from one stopover site to the next was calculated as the geodesic distance between two coordinates (centroid point of the two stopover sites) on the ellipsoid based on the WGS84 coordinate system using Vincenty’s inverse calculation ([Vincenty 1975](#_ENREF_11)). The total flight distance of each geese during spring migration was defined as the sum of the travel distances between successive stopover sites from the wintering site to their breeding site.

Reference

Ackerman, J.T., Takekawa, J.Y., Orthmeyer, D.L., Fleskes, J.P., Yee, J.L., Kruse, K.L., 2006. Spatial use by wintering greater white-fronted geese relative to a decade of habitat change in California's Central Valley. Journal of Wildlife Management 70, 965-976.

Bustnes, J., Mosbech, A., Sonne, C., Systad, G., 2010. Migration patterns, breeding and moulting locations of king eiders wintering in north-eastern Norway. Polar Biology 33, 1379-1385.

Drent, R.H., Fox, A.D., Stahl, J., 2006. Travelling to breed. Journal of Ornithology 147, 122-134.

Horne, J.S., Garton, E.O., Krone, S.M., Lewis, J.S., 2007. Analyzing Animal Movements Using Brownian Bridges. Ecology 88, 2354-2363.

Johnson, W.P., Schmidt, P.M., Taylor, D.P., 2014. Foraging flight distances of wintering ducks and geese: a review. Avian Conservation and Ecology 9, 2.

Kranstauber, B., Kays, R., LaPoint, S.D., Wikelski, M., Safi, K., 2012. A dynamic Brownian bridge movement model to estimate utilization distributions for heterogeneous animal movement. Journal of Animal Ecology 81, 738-746.

Kulldorff, M., 2006. SaTScan user guide., Boston, USA.

Kulldorff, M., Heffernan, R., Hartman, J., Assuncao, R., Mostashari, F., 2005. A space-time permutation scan statistic for disease outbreak detection. Plos Medicine 2, 216-224.

Palm, E.C., Newman, S.H., Prosser, D.J., Xiao, X., Ze, L., Batbayar, N., Balachandran, S., Takekawa, J.Y., 2015. Mapping migratory flyways in Asia using dynamic Brownian bridge movement models. Movement ecology 3, 3.

Si, Y., Skidmore, A.K., Wang, T., Boer, W.F.d., Toxopeus, A.G., Schlerf, M., Oudshoorn, M., Zwerver, S., Jeugd, H.v.d., Exo, K.-M., Prins, H.H.T., 2011. Distribution of Barnacle Geese *Branta leucopsis* in relation to food resources, distance to roosts, and the location of refuges. ARDEA 99, 217-226.

Vincenty, T., 1975. Direct and inverse solutions of geodesics on the ellipsoid with application of nested equations. Survey review 23, 88-93.
